# Impact of angiotensin-converting enzyme inhibition on hemodynamic and autonomic profile of elastase-2 knockout mice

**DOI:** 10.1590/1414-431X2022e11774

**Published:** 2022-03-21

**Authors:** T.C. Prates-Costa, M. de Oliveira, R. Fazan, H.C. Salgado, C. Becari

**Affiliations:** 1 Departamento de Fisiologia, Faculdade de Medicina de Ribeirão Preto, Universidade de São Paulo, Ribeirão Preto, SP, Brasil; 2 Departamento de Cirurgia e Anatomia, Faculdade de Medicina de Ribeirão Preto, Universidade de São Paulo, Ribeirão Preto, SP, Brasil

**Keywords:** ACE, Elastase-2, ELA-2 knockout mice, Blood pressure, Heart rate variability

## Abstract

Elastase-2 (ELA-2) is an angiotensin II-generating enzyme that participates in the cardiovascular system. ELA-2 is involved in hemodynamic and autonomic control and is upregulated in myocardial infarction and hypertension. The inhibition of angiotensin-converting enzyme (ACE) increased ELA-2 expression in the carotid arteries and heart of spontaneously hypertensive rats. In this study, we sought to investigate the role of ACE inhibition in hemodynamic and autonomic balance in elastase-2 knockout (ELA-2 KO) mice. Male ELA-2 KO and C57BL/6 mice were treated with the ACE inhibitor enalapril or saline for 10 days. After treatment, mice underwent surgery for cannulation of the femoral artery and arterial pressure recordings were made five days later in awake animals. The variability of systolic blood pressure (SBP) and pulse interval (PI) was evaluated in the time and frequency domain. Spontaneous baroreflex was assessed by the sequencing method. ACE inhibition caused a significant decrease in mean arterial pressure (117±2.2 *vs* 100±2.8 mmHg) and an increase in heart rate (570±32 *vs* 655±15 bpm) in ELA-2 KO mice. Despite a tendency towards reduction in the overall heart rate variability (standard deviation of successive values: 7.6±1.1 *vs* 4.7±0.6 ms, P=0.08), no changes were found in the root of the mean sum of squares or in the power of the high-frequency band. ACE inhibition did not change the spontaneous baroreflex indices (gain and baroreflex effectiveness index) in ELA-2 KO mice. Altogether, this data suggested that ACE played a role in the maintenance of hemodynamic function in ELA-2 KO mice.

## Introduction

Several aspects of the renin-angiotensin system (RAS) have been extensively investigated, particularly its role in cardiovascular functions and homeostasis. Considered the main active RAS peptide, angiotensin II (ANG II) has been shown to enhance sympathetic flow, arterial blood pressure, vascular contractility, and attenuation of the baroreceptor reflex (1-4).

The importance of alternative pathways for angiotensin-converting enzyme (ACE) linked to ANG II production has been demonstrated, and these include chymase (5) and elastase-2 (ELA-2) (6,7).

ELA-2 is a chymotrypsin-like serine-protease belonging to the CELA-2A family, related to ANG II yielding (6). It is found in several tissues such as the aorta and mesenteric arteries, heart, lungs, kidneys, liver, and brain (6-9). Becari and colleagues described a relationship between ELA-2 and ACE, providing evidence that the inhibition of the latter enzyme increases ELA-2 expression in the carotid arteries and hearts of Wistar rats and spontaneously hypertensive rats (SHR) (10).

In a previous study, our group described the influence of ELA-2 on the autonomic profile of mice. ELA-2 knockout (ELA-2 KO) mice presented a lower heart rate (HR) compared with their wild-type counterparts. This strain shows changes in the autonomic balance, displaying increased parasympathetic flow, possibly due to the reduction of ANG II concentrations (11). However, the participation of ACE on the autonomic and hemodynamic profile of ELA-2 KO mice is still unknown. Thus, we sought to investigate the role played by ACE inhibition in the hemodynamic and autonomic function in ELA-2 KO mice.

## Material and Methods

### Animals

Male recessive homozygous ELA-2 KO (CELA-2aTm1Bdr) and C57BL/6 mice were supplied, respectively, by Dr Helio C. Salgado's Laboratory and the Ribeirão Preto Medical School Animal Facility of the University of São Paulo (Ribeirão Preto, SP, Brazil). Mice remained in ventilated racks, under a controlled temperature of 22±1°C, 12-h light/dark cycle, with free access to water and food. All protocols were approved by the Animal Experimental Ethics Committee of the Ribeirão Preto Medical School-USP (210/2017).

### Genotyping

All mice used in this study were genotyped. Genomic DNA extracted from tissue samples (ears and tails, 1 to 2 mm) were used to perform the genotyping. The protocol consisted of adding 200 μL of NaOH (50 mM) to the samples, followed by incubation (10 min at 95°C) in a dry bath, and then the addition of 20 µL of Tris-HCl buffer (1 M, pH 8.0). The samples were centrifuged (18,227 *g* for 5 min at 4°C), and the supernatant was collected for amplification of target genes by a multiplex-PCR protocol. Three different primers, ELA2F (5′AGAAACTATGTCTGCTATGTCAC3′), Ela2WTr (5′TTTACAGATGAGGAAGTCACC3′), and ELAloxr2 (5′TTCTTGAACTGATGGCGAGC3′), were used to simultaneously evaluate the presence of the wild (+/+), heterozygous (+/-), and modified (-/-) allele. The PCR reactions consisted in 5 μL of REDextract-N-Amp, 1.2 μL of ultra-pure water, 0.6 μL of each oligonucleotide (10 mM), and 2 μL of genomic DNA (100 ng/μL) per reaction. The thermal cycling (Veriti Thermal Cycler, Applied Biosystems, USA) process had three stages consisting of 1 cycle of 3 min at 95°C, 40 cycles of amplification, in which each cycle consisted of 95°C for 30 s, 60°C for 40 s, and 72°C for 45 s, and 5 min at 72°C after the last amplification cycle. Aliquots (5 μL) of each amplified sample were processed and analyzed by electrophoresis on a 3% agarose gel containing Sybr Safe, and the molecular weight of the PCR products were compared to a 100-bp molecular weight marker. Visualization of the cDNA took place after exposure of the agarose gel to ultraviolet light to detect amplified products with previously known sizes. The possible results obtained with genotyping were: knockout mice (-/-, 295 bp amplicon), heterozygous (+/-, 245 bp and 295 bp amplicon), and wild-type (+/+, WT, 245 bp).

### Treatment

Mice were divided into four groups that received saline (0.9% saline solution) or enalapril (ACE inhibitor, 15 mg·kg^-1^·day^-1^, Sigma Aldrich, USA) given orally by gavage for 10 days. The mice were then cannulated on the 5th day and hemodynamic recording was conducted on the 10th day, as described in the next section.

### Cardiovascular assessment

The mice were anesthetized with an inhaled anesthetic (isoflurane, 5% for induction and 1.5-2% for maintenance) and kept at a controlled temperature (22±1°C). All surgical procedures were performed using a surgical microscope under aseptic conditions. The right femoral artery was cannulated using catheters manufactured with Micro-Renathane (Braintree Scientific Inc., USA) and filled with heparinized saline solution for blood pressure (BP) recording. Another catheter was implanted into the jugular vein for drug administration. The catheters were exteriorized between the shoulder blades of the mice and the incisions were sutured. After surgery, the animals received antibiotics (24,000 IU/kg, *im*, Pentabiótico Veterinário, Zoetis, Brazil) and were kept in individual cages for postoperative recovery for 5 days. About 30 min before the beginning of the experiments, the mice were taken to a noise-free room to minimize stress, and the arterial catheter was connected to a pressure transducer to record pulsatile BP. The electrical signal was amplified and sent to a computer connected to the PowerLab registration system (ADInstruments, New Zealand). Basal BP was continuously sampled (4 kHz) over 30 to 40 min.

### Evaluation of ACE inhibition efficacy

A test was performed to evaluate the ACE inhibition efficacy. The protocol was carried out involving the administration of 10 μL of ANG I (solution 1 μg/mL, Sigma Aldrich), *in bolus,* through the venous catheter, followed by ANG II administration (10 μL, solution 1 μg/mL, Sigma Aldrich) after BP had returned to baseline values.

### Heart rate variability

BP recordings were analyzed by a computer software that detects inflection points from the signals generated by the recording system (Blood Pressure Module for LabChart 7.0, AD Instruments, Australia). Time series beat-to-beat, systolic blood pressure (SBP), and pulse interval (PI) were generated using consecutive SBP values. All time series were obtained from baseline recordings (30-40 min). Autonomic, cardiac, and BP modulations were investigated by analyzing heart rate variability (HRV) and SBP variability in the time and frequency domain. SBP variability was quantified in the time domain by the standard deviation of successive BP values (SD). The PI variability was quantified by the standard deviation of successive values (SDNN) and the square root of the mean sum of squares of the differences between successive PI values (RMSSD). The variability of SBP and PI was also examined in the frequency domain by spectral analysis. Beat-to-beat series of SBP and PI were interpolated at 10 Hz and divided into continuous 512 beat segments, overlapping by 50%. Each segment was subjected to a Hanning window and had its spectra calculated by the Fast Fourier Transform (FFT) using the customized computer software CardioSeries v2.4 (http://www.danielpenteado.com/cardioseries). The oscillatory components found were quantified at low (LF: 0.20 to 1.0 Hz) and high frequency (HF: 1.0 to 5.0 Hz) bands (12).

### Baroreflex assessment

Spontaneous baroreflex function was evaluated using the sequence technique and also using CardioSeries. First, pressure ramps, ascending or descending with a minimum duration of 4 consecutive beats, were identified in the SBP series. Then, the software seeks PI ramps (in the same direction), displaced by one cardiac beat (delay=1) whose linear correlations with pressure ramps are greater than 0.8. When this occurred, a spontaneous baroreflex sequence was identified and the angular coefficient of each regression was used as the spontaneous baroreflex sensitivity (gain). In addition, the baroreflex effectiveness index (BEI) was calculated by the ratio between the number of sequences and the total number of BP ramps. The BEI is interpreted as the percentage of BP ramps, which effectively produces a baroreflex response (13).

### Statistical analysis

Data are reported as means±SE. The effects of ACE inhibition between the C57BL/6 and ELA-2 KO groups were examined by two-way ANOVA. The significance level adopted was P<0.05. GraphPad Prism 7.0 (USA) was used for statistical analysis and graphing.

## Results

### ACE inhibition efficacy from the responses of the mean arterial pressure

**Figure 1:**
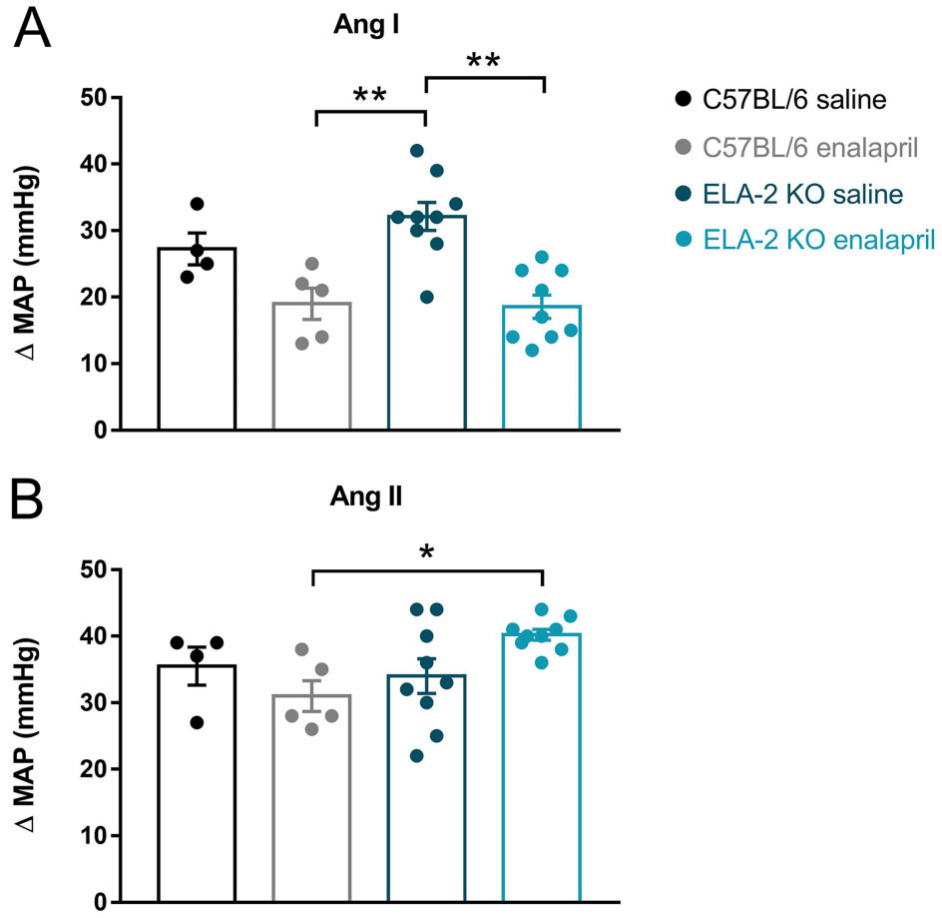
Mean arterial pressure variation (ΔMAP) after angiotensin I (Ang I) (**A**) and angiotensin II (Ang II) (**B**) administration in knock-out mice (ELA-2 KO) and wild-type mice (C57BL/6) treated with saline or enalapril. Data are reported as means±SE. **P<0.005, *P<0.05, two-way ANOVA.

Enalapril treatment diminished the vasopressor response (mean arterial pressure difference (ΔMAP)) to ANG I in the ELA-2 KO mice by approximately 41% compared with the littermates treated with saline (19±1.8 *vs* 32±2.1 mmHg in the saline group, P=0.0002). However, ACE inhibition did not change the C57Bl/6 response to ANG I (19±2.4 *vs* 27±2.4 in saline-treated animals, P=0.15). As shown in Figure 1, the MAP response to ANG II was more significant in the enalapril-treated ELA-2 KO group than in the C57BL/6 group, which received the same treatment (40±0.8 *vs* 31±2.3, P=0.04).

### Effect of enalapril on the MAP and HR

**Figure 2:**
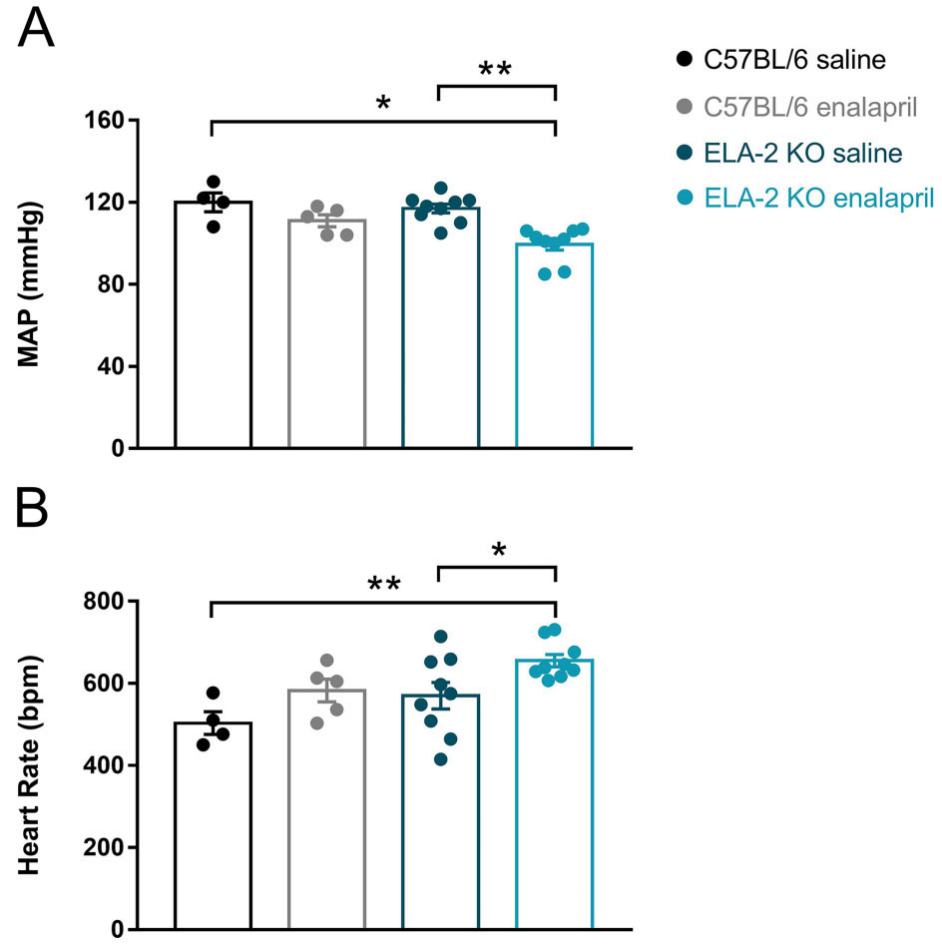
Basal mean arterial pressure (MAP) and heart rate of knock-out mice (ELA-2 KO) and wild-type mice (C57BL/6) treated with saline or enalapril. **A**, Enalapril treatment for 10 days significantly reduced ELA-2 KO mice MAP compared to the saline group. **B**, Heart rate was slightly increased after 10 days of treatment with the ACE inhibitor. Data are reported as means±SE. **P<0.005, *P<0.05, two-way ANOVA.

Enalapril treatment significantly reduced MAP of ELA-2 KO mice (100±1.0 *vs* 117±2.2 mmHg in the saline group, P=0.0012), but no change was observed in the C57Bl/6 group (111±3.0 *vs* 120±5.5 in the saline group, P=0.85). Enalapril did not promote changes in C57BL/6 HR (583±27.7 *vs* 503±27.5 in the saline group, P=0.61). However, an increase in HR was observed after ACE inhibitor treatment on ELA-2 KO mice compared to the saline-treated littermates (655±15.1 *vs* 570±32.1 bpm in the saline group, P=0.01), as shown in Figure 2.

### Effect of enalapril on SBP variability

**Figure 3:**
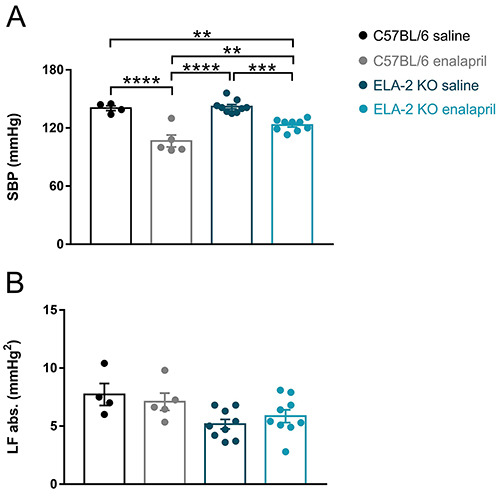
Systolic blood pressure (SBP) and low frequency (LF) band power of knock-out mice (ELA-2 KO) and wild-type mice (C57BL/6) treated with saline or enalapril. **A**, SBP after 10 days of saline or enalapril treatment. **B**, SBP low frequency band power. Data are reported as means±SE. **P<0.01, ***P<0.001, ****P<0.0001, two-way ANOVA. abs: absolute values.

ACE inhibition with enalapril reduced SBP in both studied strains (ELA-2 KO: 123±1.9 *vs* 142±2.2 mmHg in the saline group, P=0.002; C57Bl/6: 107±6.2 *vs* 141±2.5 mmHg in the saline group). The reduction was significant in C57BL/6 mice compared to ELA-2 KO mice (123±1.9 *vs* 107±6.2 mmHg, saline groups P=0.007). As shown in Figure 3 no difference was observed in the low frequency (LF) band power among groups.

### Effect of enalapril on HR and SBP variability

The frequency domain analysis showed that ELA-2 KO mice presented an attenuated HRV compared to C57BL/6 with regard to the parameters presented in absolute values: very low frequency (VLF) (8.3±1.4 *vs* 20.2±2.7 ms, P=0.001), LF (7.5±1.4 *vs* 14.6±0.8 ms, P=0.003), and HF (3.3±0.8 *vs* 8.6±0.8 ms, P=0.0003). Also, ACE inhibition provoked a reduction in the observed parameters between the C57BL/6 groups: VLF (3.9±0.1 *vs* 20.2±2.7 ms in the saline group, P<0.0001), LF (3.6±1.0 *vs* 14.6±6 ms, P=0.0001), and HF (1.2±0.2 *vs* 8.6±0.8, P<0.0001). In ELA-2 KO mice, enalapril treatment only reduced the LF band power in absolute values (3.5±0.6 *vs* 7.5±1.4 ms in the saline group, P=0.04). The LF/HF ratio was similar among the studied groups. In the time domain, no differences were found in RMSSD, but a reduction was seen in the SDNN after ACE inhibition in C57BL/6 mice (5.3±1.1 *vs* 10.2±0.7 ms in the saline group, P=0.02), possibly due to a decrease in the global variability. Despite this, the SDNN of ELA-2 KO mice was not significantly affected after ACE inhibition, but a tendency of attenuation of this parameter was observed (4.7±0.6 *vs* 7.6±1.1 ms in the saline group, P=0.08) (Table 1).

**Table 1:** Pulse interval and systolic blood pressure variability of C57BL/6 and ELA-2 knock-out (KO) mice 10 days after treatment with enalapril or saline.

	C57BL/6		ELA-2 KO	
	Saline	Enalapril	Saline	Enalapril
Pulse interval variability				
SDNN, ms	10.2±0.7	5.3±1.1*	7.6±1.1	4.7±0.6**
RMSSD, ms	5.2±0.3	2.8±0.7	4.2±0.8	2.4±0.3
VLF, ms	20.2±2.7	3.9±0.1*	8.3±1.4**	4.8±0.9***
LF, ms^2^	14.6±0.8	3.6±1.0***	7.5±1.4**	3.5±0.6****^#^
HF, ms^2^	8.6±0.8	1.2±0.2****	3.3±0.8***	1.8±1.4****
LF, nu	66±2.8	59±1.3	67±1.8	64±4.0
HF, nu	34±2.8	41±1.3	33±1.8	36±4.0
LF/HF	2.3±0.2	2.0±0.1	2.5±0.2	2.5±0.4
Systolic blood pressure variability				
SDNN, mmHg	4.3±0.3	4.1±0.2	4.9±0.2	4.5±0.3
LF, mmHg	7.7±0.9	7.1±0.7	5.2±0.4	5.9±0.6

Data are reported as means±SE. *P<0.05, **P<0.005, ***P<0.001, ****P<0.0001 compared with C57BL/6 saline group; ^#^P<0.05 compared with ELA-2 KO saline group (two-way ANOVA). SDNN: standard deviation of successive values; RMSSD: root of the mean sum of squares of the differences; VLF: very low frequency band power; LF: low frequency; HF: high frequency.

### Effect of enalapril on spontaneous baroreflex

**Figure 4:**
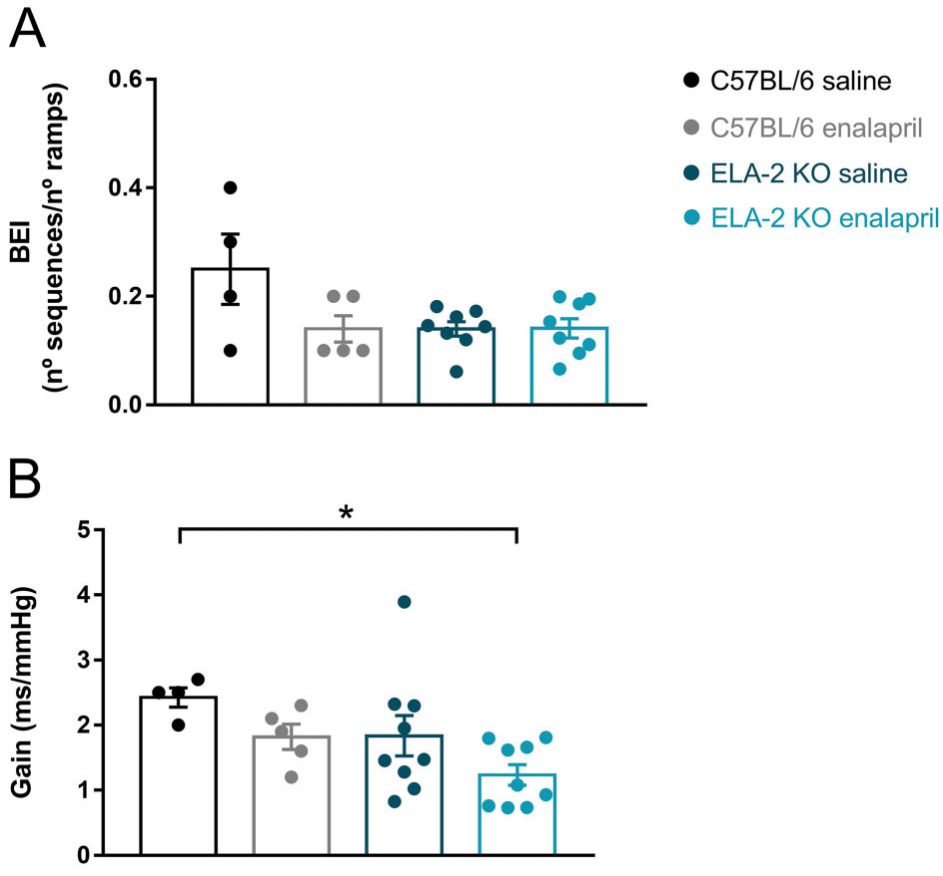
**A**, Baroreflex effectiveness index (BEI). **B**, Baroreflex gain of knock-out mice (ELA-2 KO) and wild-type mice (C57BL/6) treated with saline or enalapril. Data are reported as means±SE. *P<0.05, two-way ANOVA.

ACE inhibition with enalapril did not affect the BEI among the groups (ELA-2 KO: 0.14±0.01 *vs* 0.14±0.01 in the saline group; C57BL/6: 0.14±0.02 *vs* 0.25±0.06 in the saline group). Also, as shown in Figure 4, no differences were observed in baroreflex gain of the studied groups (ELA-2 KO: 1.2±0.2 *vs* 1.8±0.3 in the saline group; C57BL/6: 1.8±0.2 *vs* 2.4±0.2 in the saline group).

## Discussion

The data obtained in the present study suggested that ACE played an important role in BP, HR, and HRV maintenance in ELA-2 KO mice. Although ACE inhibition did not seem to result in significant alterations in BP variability, a tendency for HRV reduction was seen (Table 1).

ACE inhibitors have been used for decades as a treatment for arterial hypertension and they improve outcomes in heart failure and chronic renal patients by decreasing ANG II levels (14-16). However, in the long term, patients treated with ACE inhibitors may present unchanged ANG II concentrations (17,18), possibly because of the action of ACE-alternative enzymes in ANG II production, i.e., chymase (5) and ELA-2 (6).

A previous study showed that ELA-2 KO promotes reduction in HR, changing the sympathovagal balance in mice and enhances the parasympathetic activity without affecting BP (11). ACE inhibition in ELA-2 KO mice caused a significant decrease in BP (Figure 2) as expected, probably due to the withdrawal of two important routes of ANG II production. This hypothesis is based on the importance of the RAS and tissue ANG II in the modulation of the BP (19). Also, a BP reduction was not observed in C57BL/6 mice, possibly indicating the importance of ELA-2 for hemodynamic modulation.

Increased HR was found in ELA-2 KO mice after enalapril treatment, but HR was not significantly altered in C57BL/6 animals (Figure 2). This result would be expected as a baroreflex (the major BP control mechanism) response under physiological conditions, where a BP reduction would result in a compensatory HR increase. However, 10 days after the first dose of enalapril, it adjusted to a new BP set-point and the HR returned to the baseline (20,21).

Despite C57BL/6 and ELA-2 KO mice having similar BEI values, a reduction was seen in the baroreflex gain of the ELA-2 KO enalapril group compared to the C57BL/6 saline group (Figure 4). It is opportune to highlight the lack of information about the impact of ACE-inhibitors on BP and HR variability and how different types of drugs can impact these variables in normotensive subjects.

In this investigation, no change was observed in BP variability of ELA-2 KO or C57BL/6 mice after ACE inhibition. This outcome was represented by the power of the LF band (Figure 3, Table 1), a reliable parameter of sympathetic activity, since blood vessels receive mainly sympathetic innervation. On the other hand, enalapril treatment reduced global HRV in C57BL/6 mice, corroborating previous reports, which stated that both captopril and enalapril can reduce global HRV (22,23). Interestingly, these studies also observed that HR increases after ACE-inhibitor treatment, as seen in ELA-2 KO mice, which is unexpected since human studies suggest that HR is usually not affected with therapeutic doses of captopril (24).

The present research also found a reduction in HRV in ELA-2 KO mice compared to C57BL/6 mice (Table 1). Nonetheless, HRV did not change in ELA-2 KO mice after enalapril treatment, except for a small reduction in the LF power band (absolute values, ms). The PI LF band can reflect both parasympathetic and sympathetic activity. However, since a reduction was not seen for the other parameters related to the autonomic activity evaluated, it is unlikely that an activity reduction occurred after ACE inhibition. Thus, this phenomenon requires further investigation.

This is the first study investigating the impact of the ACE on the hemodynamic and autonomic profile of ELA-2 KO mice. Altogether, the data suggested that ACE was required for BP and HR maintenance in this ELA-2 KO mouse model. The precise mechanism(s) underlying the interplay between ACE and ELA-2 for BP and HR control in mice remains unknown.
